# Serum levels of interleukin-6 are linked to the severity of the disease caused by Andes Virus

**DOI:** 10.1371/journal.pntd.0005757

**Published:** 2017-07-14

**Authors:** Jenniffer Angulo, Constanza Martínez-Valdebenito, Claudia Marco, Héctor Galeno, Eliecer Villagra, Lilian Vera, Natalia Lagos, Natalia Becerra, Judith Mora, Andrea Bermúdez, Janepsy Díaz, Marcela Ferrés, Marcelo López-Lastra

**Affiliations:** 1 Laboratorio de Virología Molecular, Instituto Milenio de Inmunología e Inmunoterapia (IMII), Departamento de Enfermedades Infecciosas e Inmunología Pediátrica, División de Pediatría, Escuela de Medicina, Pontificia Universidad Católica de Chile, Santiago, Chile; 2 Laboratorio de Infectología, Departamento de Enfermedades Infecciosas e Inmunología Pediátrica, División de Pediatría, Escuela de Medicina, Pontificia Universidad Católica de Chile, Santiago, Chile; 3 Subdepartamento de Virología Clínica, Departamento Laboratorio Biomédico Nacional y de Referencia, Instituto de Salud Pública de Chile, Santiago, Chile; 4 Departamento de Asuntos Científicos, Instituto de Salud Pública de Chile, Santiago, Chile; University of Texas Medical Branch, UNITED STATES

## Abstract

Andes virus (ANDV) is the etiological agent of hantavirus cardiopulmonary syndrome in Chile. In this study, we evaluated the profile of the pro-inflammatory cytokines IL-1β, IL-12p70, IL-21, TNF-α, IFN-γ, IL-10 and IL-6 in serum samples of ANDV-infected patients at the time of hospitalization. The mean levels of circulating cytokines were determined by a Bead-Based Multiplex assay coupled with Luminex detection technology, in order to compare 43 serum samples of healthy controls and 43 samples of ANDV-infected patients that had been categorized according to the severity of disease. When compared to the controls, no significant differences in IL-1β concentration were observed in ANDV-infected patients (p = 0.9672), whereas levels of IL-12p70 and IL-21 were significantly lower in infected cases (p = <0.0001). Significantly elevated levels of TNF-α, IFN-γ, IL-10, and IL-6 were detected in ANDV-infected individuals (p = <0.0001, 0.0036, <0.0001, <0.0001, respectively). Notably, IL-6 levels were significantly higher (40-fold) in the 22 patients with severe symptoms compared to the 21 individuals with mild symptoms (p = <0.0001). Using multivariate regression models, we show that IL-6 levels has a crude OR of 14.4 (CI: 3.3–63.1). In conclusion, the serum level of IL-6 is a significant predictor of the severity of the clinical outcome of ANDV-induced disease.

## Author summary

Andes virus (ANDV) causes hantavirus cardiopulmonary syndrome (HCPS) that is characterized by the development of vascular leakage syndrome, eventually leading to massive pulmonary edema, shock and, in many cases, death. To date, no FDA-approved immunotherapeutics, specific antivirals, or vaccines are available for use against HCPS. Patient survival rates hinge largely on early virus diagnosis, hospital admission and aggressive pulmonary and hemodynamic support in an intensive care unit. Individual host factors associated with the outcome of an ANDV infection are poorly known, and such knowledge could allow the disease progression of hospitalized patients to be predicted, resulting in individualized treatment. In this study, we show that serum levels of IL-6 at the time of hospitalization in ANDV-infected patients are associated with the severity of the clinical outcome of ANDV-induced disease. Therefore, these finding suggest that determining IL-6 levels at the time of admission to the hospital could be useful to predict the progression of ANDV-induced disease.

## Introduction

Andes virus (ANDV) is a rodent-borne hantavirus member of the *Bunyaviridae* family, and is unique for its ability to be transmitted from person-to-person [1,2]. ANDV is endemic in Chile and according to the Chilean Ministry of Health (Department of Epidemiology, Chilean Ministry of Health; http://www.minsal.cl), 1087 cases of ANDV infection have been confirmed through May 2017 with a lethality rate of 35–40%. In humans, ANDV infection causes hantavirus cardiopulmonary syndrome (HCPS) [3,4]. The initial symptoms of HCPS are non-specific and include fever, headache, and myalgia among others. However, in later phases, symptoms progress from coughing to severe pulmonary edema requiring intubation and mechanical ventilation, whilst cardiogenic shock is the main cause of death [3,4]. Like other hantaviruses, the ANDV-induced illness is associated with the activation of the host's innate immune response, with cytokines playing a key role, rather than with direct cellular destruction induced by active virus replication [5]. Currently, no FDA approved drugs, immunotherapeutics, or vaccines are available for HCPS prevention or treatment [6]. Thus, patients’ survival rates hinge largely on early diagnosis, hospital admission and aggressive pulmonary and hemodynamic support in an intensive care unit [7,8]. Moreover, there are no blood biomarkers to predict the outcome of ANDV-induced HCPS. Interestingly, several reports show that the levels of the pro-inflammatory cytokines TNF-α, IL-1, IL-6, IL-10, and IFN-γ increase in patients infected with other hantaviruses such as Puumala Virus (PUUV), Dobrava Virus (DOBV), or the Sin Nombre Virus (SNV) [5,9,10,11,12,13,14,15]. Additionally, high levels of IL-6 and TNF-α in plasma of SNV- and PUUV-infected patients is associated with a severe or fatal disease outcome [5,10,13,14,16]. In the case of ANDV, elevated levels of cytokines including IL-6 were reported in ANDV-infected air-exposed organotypic human lung tissues [17]. Motivated by these findings, we designed a study to evaluate the cytokine profile (IL-1β, IL-12p70, IL-21, TNF-α, IFN-γ, IL-10, IL-6) in serum samples of Chilean ANDV-infected patients collected at the time of hospitalization with the aim of establishing if the levels of any of the selected cytokines are linked to the severity of ANDV-induced disease. Cytokine selection was based on reports published for ANDV and other hantaviruses [5,10,13,14,16]. Three cohorts were included; a group of healthy controls, a cohort of ANDV-infected patients with mild disease progression, and a cohort of ANDV-infected patients with a severe disease progression, many of whom subsequently died. Results show that on admission to hospital TNF-α, IFN-γ, IL-10, and IL-6 levels were elevated in serum of ANDV-infected patients compared to controls. Importantly, the serum levels of IL-6 in ANDV-infected patients at the time of hospitalization were associated with the severity of the clinical outcome of ANDV-induced disease.

## Materials and methods

### Study population

A total of 43 non-heat inactivated serum samples from ANDV-infected patients were selected based on their availability. Samples were obtained from a collection generated between January 2006 and January 2014 and stored at the Instituto de Salud Pública (ISP) de Chile or at the Laboratorio de Infectología, Facultad de Medicina, Pontificia Universidad Católica de Chile. These samples were collected for ANDV diagnosis at the time the patient was admitted to hospital, equivalent to 2–11 days after suspected ANDV infection (prodromic stage of the clinical course of HCPS). After collection, samples were stored at -8°C and not thawed more than once before use. In a previous study, the samples used had been catalogued as coming from patients with mild or severe symptoms, according to the severity of the final outcome of the ANDV-induced disease [18]. Selected patients were non-related individuals and were considered to be representative of each group. Mild hantavirus infection was characterized by a febrile illness with unspecific symptoms such as headache, myalgias, chills, gastrointestinal symptoms, and no or minimal respiratory compromise [18]. Severe cases exhibited rapid and progressive impaired lung function with the need for an external oxygen supply and the use of vasoactive drugs, resulting in shock and/or death [18]. Based on this categorization, of the 43 infected samples used in this study, 21 were from mild casesand 22 were from severe cases. Fourteen individuals from the latter group subsequently died of HCPS, whilst there were no fatalities in the group showing mild symptoms. Approval for the use of the samples in this study was obtained from the Ethical Review Board of the Facultad de Medicina, Pontificia Universidad Católica de Chile (code 12–292 and 14–438). ANDV infection was confirmed by positive hantavirus immunoglobulin (IgM) serology or by ANDV genome detection by reverse transcription polymerase chain reaction (RT-PCR) [18,19,20]. Control samples correspond to 43 non-heat treated serum samples obtained from healthy donors, who tested negative for ANDV, the human immunodeficiency virus, and hepatitis B and C virus infection.

### Determination of cytokine levels in serum samples

Cytokines IL-1β, IL-12p70, IL-21, TNF-α, IFN-γ, IL-10 and IL-6 were measured in serum samples using a custom made Milliplex magnetic bead panel (Merck KGaA, Darmstadt, Germany) following the manufacturer’s instructions. The lower limit of detection (pg/mL) for each cytokine in the assay was: IL-1β, 2.5; IL-12p70, 1.84; IL-21, 2.37; TNF- α, 1.23; IFN-γ, 4.95; IL-10, 4.5 and IL-6, 3.34.

### Statistical analysis

Results were analyzed using Graph Prism V6.0 (La Jolla, CA, USA) and Statistical Package for the Social Sciences (SPSS) V10.1 (SPSS, Inc., Chicago, IL, USA) software. The significance between the clinical and laboratory findings variables, and the clinical outcome was calculated by a Fisher`s exact test. The relationship between the clinical outcome and the cytokine levels was determined by a Mann-Whitney test for continuous variables using only two variables in each analysis. The crude odd ratio (OR) values were calculated by a univariate logistic regression analysis followed by a multivariate stepwise forward and reverse logistic regression analysis using SPSS V10.1 software package. P values of <0.05 were considered as significant.

## Results

The general characteristics of the ANDV-infected patients included in the study are summarized in Table 1. The mean age of the patients was 33 ± 14 years and 60.5% were male (Table 1). The vast majority of patients (97.7%) were infected between 32° 02’ and 56° 30’ south latitude (in the center and south of Chile) in rural areas (92.5%), consistent with the geographical distribution of the known viral reservoir in the long-tailed pygmy rice rat (*Oligoryzomys longicaudatus*) [21]. The main clinical features and laboratory findings of the ANDV-infected patients, at the time of their admission in a hospital care unit are summarized in Table 2. In a previous study, samples of ANDV-infected patients used in this study had been grouped into mild or severe cases depending on the final clinical outcome of the disease [18] (Table 1). A univariante analysis revealed that respiratory distress and haematocrit levels at the time of sample collection were significantly higher in patients that ultimately exhibited a severe clinical outcome (Table 2).

**Table 1:** Characteristic of andes virus-infected patients (n = 43).

Variable	Patients, No. (%)
**Sex**	
Male [n (%)]	26 (60.5)
Female [n (%)]	17 (39.5)
**Age (mean, ± SD)**	33 ±14
0–15 [n (%)]	3 (7.0)
16–30 [n (%)]	15 (34.9)
31–50 [n (%)]	19 (44.1)
51–80 [n (%)]	6 (14.0)
**Area (South latitude)**	
North (17º30’ to 32º16’) [n (%)]	1 (2.3)
Center (32º02’to 36º33’) [n (%)]	8 (18.6)
South (36º00’ to 56º30’) [n (%)]	34 (79.1)
Area of infection [n (%)]^a^	
Urban area	3 (7.5)
Rural area	37 (92.5)
**Outcome**	
Mild [n (%)]	21 (48.8)
Severe [n (%)]^b^	22 (51.2)
Total Lethality [n (%)]	14 (32.5)

Abbreviation: SD, Standard deviation.

^a^ 40 patients with available data.

^b^ Including the 14 deceased patients.

**Table 2:** Available clinical and laboratory findings at the time of sample collection.

Variable	Patients, No. (%)		P value
	Severe Disease^a^	Mild Disease^a^	
**Fever (>38.5°C)**	22 (100.0)	19 (90.5)	0.2326
**Gastrointestinal symptoms**	16 (72.7)	16 (76.2)	0.5360
**Headache**	20 (90.9)	18 (85.7)	0.4771
**Myalgia**	18 (81.8)	18 (85.7)	0.5274
**Respiratory distress**	22 (100.0)	12 (57.1)	***0.0005
**Infiltrates on chest X-ray**	19 (86.4)	15 (71.4)	0.2043
**Blood shift (>10% bands)**	6 (27.3)	2 (9.5)	0.1350
**Atypical lymphocytosis**^**b**^	2 (10.5)^c^	1 (4.8)	0.4615
**Thrombocytopenia**^**d**^	19 (86.4)	19 (90.5)	0.5229
**Increased hematocrit**^**e**^	12 (54.5)	5 (23.8)	*0.0394

^a^ Classified according to clinical outcome.

^b^ Defined as >10% atypical lymphocytes.

^c^ Available data for 19 severe patients.

^d^ Defined as < 150.000 mm^3^.

^e^ Defined as >52% for men and >48% for women.

### Cytokine levels in serum of healthy controls and ANDV-infected patients

**Fig 1:**
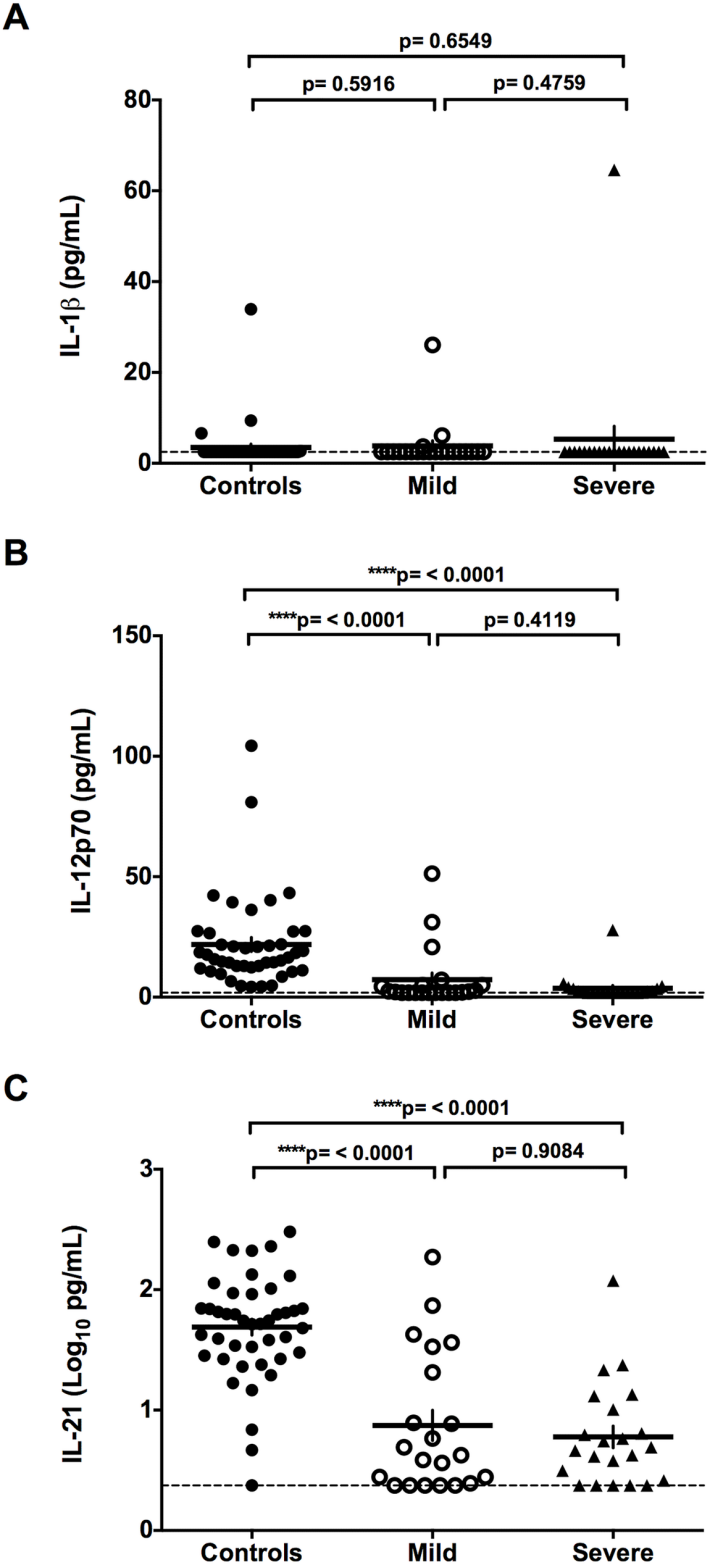
IL-1β, IL-12p70, and IL-21 levels in control and ANDV-infected patients. Circulating levels of cytokines (A) IL-1β, (B) IL-12p70 and (C) IL-21 in serum samples of Control individuals and ANDV-infected patients with Mild or Severe disease progression are represented by dots. IL-1β and IL-12p70 were expressed in pg/mL while the circulating levels of IL-21 are expressed as Log_10_ (pg/mL) to facilitate comparison between the groups. A Mann-Whitney test for continuous variables was performed to evaluate significant differences between Controls and Mild or Severe, and Severe versus Mild groups. The ends of the brackets indicate the compared groups. The dashed line represents the lower limit of detection. Each serum sample was analyzed in duplicate in the same plate.

**Fig 2:**
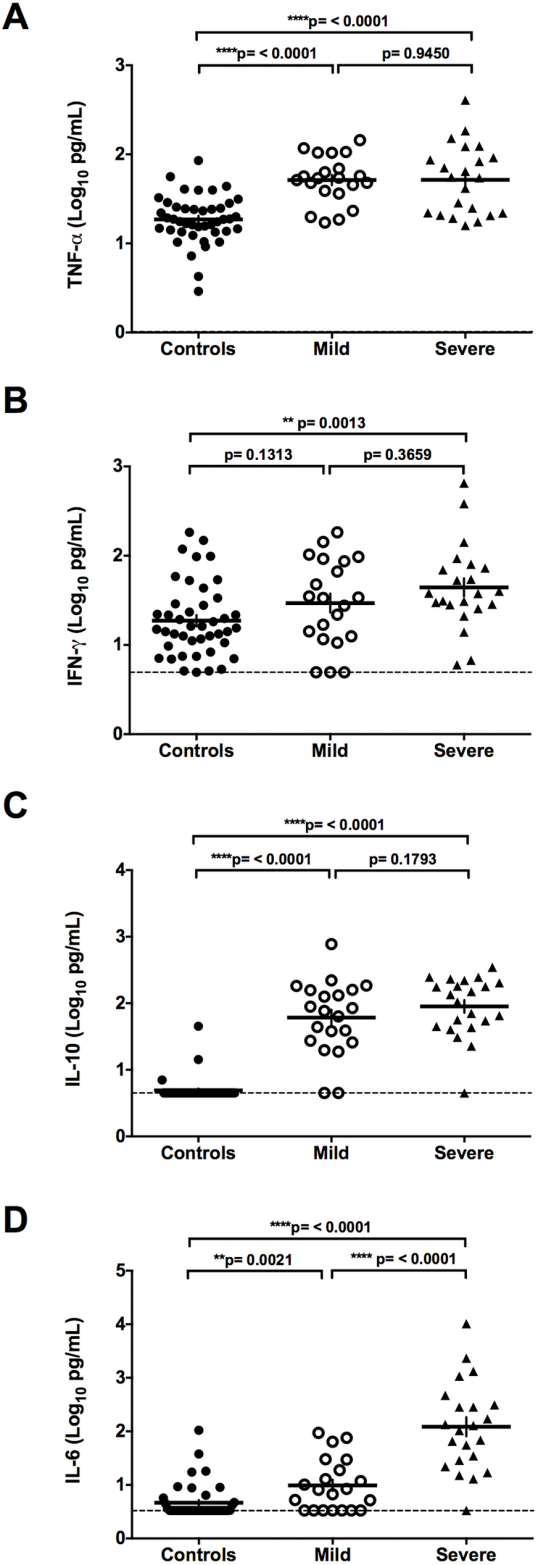
TNF-α, IFN-γ, IL-10, and IL-6 levels in control and ANDV-infected patients. Circulating levels of cytokines (A) TNF-α, (B) IFN-γ, (C) IL-10, and (D) IL-6 in serum samples of Control individuals and ANDV-infected patients with Mild or Severe disease progression are represented by dots. Each value expressed in pg/mL was transformed to Log_10_ (pg/mL) to facilitate comparison between the groups. A Mann-Whitney test for continuous variables was performed to evaluate significant differences between Controls and Mild or Severe, and Severe versus Mild groups. The dashed line represents the lower limit of detection. The ends of the brackets indicate the compared groups. Each serum sample was analyzed in duplicate in the same plate.

**Fig 3:**
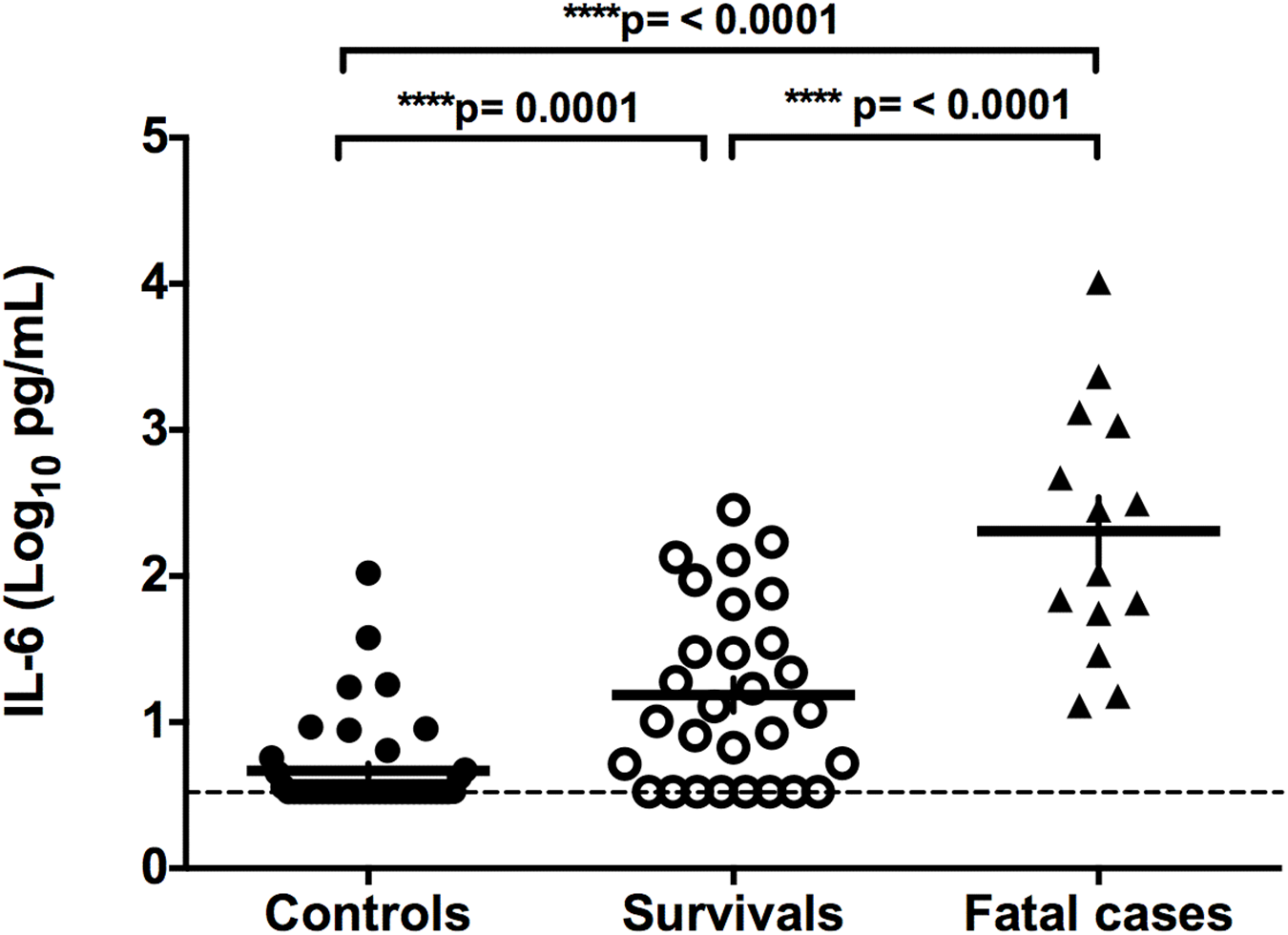
Serum levels of IL-6 in control, HCPS survivals or fatal cases. Circulating levels of IL-6 in serum samples of control individuals and HCPS survivors or fatalities are represented by dots. Values are expressed in Log_10_ (pg/mL). A Mann-Whitney test for continuous variables was performed to evaluate significant differences between controls individuals and HCPS survivors or fatalities. The ends of the brackets indicate the compared groups. The dashed line represents the lower limit of detection. Each serum sample was analyzed in duplicate in the same plate.

The levels of pro-inflammatory cytokines in serum samples of healthy controls and ANDV-infected patients were determined using a custom designed Th17 based Bead-Based Multiplex assay coupled with a Luminex platform. The results obtained for IL-1β, IL-12p70, IL-21, TNF-α, IFN-γ, IL-10, and IL-6 in each group are shown in Figs 1 and 2, whilst the mean concentration (pg/mL) of each cytokine is shown in Table 3. When compared to the group of non-infected controls, no significant differences in IL-1β concentration were observed in ANDV-infected patients (mild plus severe patients; p = 0.9672), when comparing the control group with each individual sub-group (mild p: 0.5916; severe p = 0.6549), or between those suffering mild or severe symptoms (p = 0.4759) (Fig 1A and Table 3). The levels of IL-12p70 and IL-21 were significantly lower in ANDV-infected patients when compared to the controls (p = <0.0001), although no differences in the levels of IL-12p70 (p = 0.4119) and IL-21 (p = 0.9084) were detected between those showing mild and severe symptoms (Fig 1B and 1C, and Table 3). The expression of TNF-α (Fig 2A), IFN-γ (Fig 2B), IL-10 (Fig 2C), and IL-6 (Fig 2D) exhibited higher levels in ANDV-infected individuals than in the non-infected control group, and levels were not affected by disease severity in the case of TNF-α, IFN-γ, and IL-10 (Fig 2A–2C and Table 3). Notably however, the severe group of ANDV-infected patients displayed significantly more IL-6 compared to those with milder disease symptoms (2.1 log_10_, fold increase of 40.4; p <0.0001) (Fig 2D and Table 3). Due to the evident overlap in the levels of IL-6 when patients are categorized as mild or severe, it may be that reliance on IL-6 to determine a "severe" outcome for a patient is inadequate. To address this issue, the 43 patients were grouped according to their real final status, as either survivors or fatalities (Fig 3 and Table 4). For this, the eight severe patients who survived were grouped with the 21 mild individuals (none of whom died), and these 29 patients were then compared with the 14 severe and fatal cases of ANDV infection. The results show that the serum concentration of IL-6 was significantly higher in the fatal cases compared to the surviving (2.0 log_10_, fold increase of 28.3; p <0.0001) or the control group (3.2 log_10_, fold increase 150.8; p <0.0001) (Fig 3 and Table 4).

**Table 3:** Cytokine concentration in serum of controls and ANDV-infected patients.

Cytokine	Controls [mean (min-max) pg/mL] (n = 43)	ANDV-Infected Patients [mean (min-max) pg/mL]		
		Total (n = 43)	Mild Disease^a^ (n = 21)	Severe Disease^a^ (n = 22)
**IL-1β**	3.49 (2.5–33.94)	4.60 (2.5–64.62)	3.84 (2.5–26.06)	5.32 (2.5–64.62)
**IL-12p70**	21.86 (1.84–104.31)	5.43 (1.84–51.26)	7.28 (1.84–51.26)	3.67 (1.84–27.88)
**IL-21**	74.23 (2.37–303.0)	16.75 (2.37–186.5)	21.57 (2.37–186.5)	12.14 (2.37–119.5)
**TNF-α**	21.55 (2.90–85.35)	70.05 (15.87–407)	61.01 (17.18–144.5)	78.68 (15.87–407)
**IFN-**γ	31.38 (4.95–183.0)	69.62 (4.95–652.0)	50.21 (4.95–183.0)	88.13 (5.99–652.0)
**IL-10**	5.74 (4.5–45.49)	133.21 (4.5–776.0)	129.85 (4.5–776.0)	136.25 (4.5–347.0)
**IL-6**	7.84 (3.34–105.64)	411.94 (3.34–10281.8)	19.47 (3.34–75.66)	786.57 (3.34–10281.8)

^a^ Classified according to clinical outcome.

**Table 4:** Cytokine concentration in serum of survivors or deceased ANDV-infected patients.

Cytokine	ANDV-Infected Patients [mean (min-max) pg/mL]			
	Total (n = 43)	Survivals^a^ (n = 29)	Fatal cases^a^ (n = 14)	P value (Mann-Whitney test)
**IL-1β**	4.60 (2.5–64.62)	3.48 (2.5–26.06)	6.94 (2.5–64.62)	>0.9999
**IL-12p70**	5.43 (1.84–51.26)	5.96 (1.84–51.26)	4.35 (1.84–27.88)	0.7508
**IL-21**	16.75 (2.37–186.5)	17.36 (2.37–186.5)	15.48 (2.37–119.5)	0.5314
**TNF-α**	70.05 (15.87–407)	61.75 (17.18–144.5)	87.26 (15.87–407)	0.6521
**IFN-γ**	69.62 (4.95–652.0)	81.85 (4.95–652)	44.27 (5.99–93.57)	0.8880
**IL-10**	133.21 (4.5–776.0)	128.40 (4.5–776.0)	143.22 (4.5–347.0)	0.3618
**IL-6**	411.94 (3.34–10281.8)	41.59 (3.34–285)	1179.08 (13.075–10281.8)	**** <0.0001

^a^ Classified according to clinical outcome.

### Risk factor analysis by using a logistic regression model

To control the potential confounding effects of the various risk factors identified by the univariate analysis, a multiple logistic regression model was constructed using the outcome and symptom severity of ANDV-infection, as the response variables. The stepwise forward and reverse logistic regression analysis included clinical variables (fever, gastrointestinal symptoms, headache, myalgia, respiratory distress, infiltrates on chest x-ray, blood shift, atypical lymphocytosis, thrombocytopenia, increased hematocrit) and the cytokine profiles. All cytokine values were dichotomized (above or under mean values). Table 5 summarizes the crude odd ratios (OR) for each variable processed by a univariate logistic regression model, and the OR obtained by a multivariate stepwise forward (model 1) and reverse (model 2) logistic regression analysis. In model 1, respiratory distress (p = 0.9999) and IL-6 (p = 0.0130) are the only variables that affect HCPS outcome. In model 2, respiratory distress (p = 0.9999), increased hematocrit (p = 0.0670), and IL-6 (p = 0.0150) impact on HCPS outcome. However, for both models, only the difference between the levels of IL-6 in mild and severe ANDV-infected patients is significant, with OR values greater than 1.

**Table 5:** Logistic regression of clinical and cytokine level variables.

Variable	crude OR	CI_or_	OR_model 1_ (IL-6 and respiratory distress)	CI_or_	OR_model 2_ (IL-6, respiratory distress and increased hematocrit)	CI_or_
**Fever ^a^**	1.0	-				
**Gastrointestinal symptoms**	1.2	0.3–4.7				
**Headache**	0.6	0.1–4.1				
**Myalgia**	1.3	0.3–6.8				
**Respiratory distress ^a^**	1.0	-	1.0	-	1.0	-
**Infiltrate on chest X-Ray**	0.4	0.1–1.8				
**Blood shift**	0.3	0.1–1.6				
**Atypical lymphocytosis**	2.2	0.2–26				
**Thrombocytopenia**	1.5	0.2–10				
Increased hematocrit*	3.8	1–14.2			6.9	0.8–54.9
**IL-1β**	0.4	0.4–5.4				
**IL-12p70**	0.3	0.3–3.0				
**IL-21**	0.5	0.1–2.4				
**TNF-α**	1.6	0.5–5.7				
**IFN-γ**	1.4	0.4–4.8				
**IL-10**	2.0	0.6–6.9				
IL-6*	14.4	3.3–63.1	10	1.6–74.7	10.9	1.6–74.7

^a^ OR not calculated because one or more boxes contain zero.

_*_ Significant

OR model 1: Stepwise Forward

OR model 2: Stepwise Reverse

In summary, the results from this study show that IL-6 serum concentration at the time of patient hospitalization is linked to the severity of the disease induced by ANDV.

## Discussion

A growing number of reports suggest that the hosts’ immune response plays a key role in hantavirus induced disease [5,9,10,11,12,13,14,15,22]. Studies indicate that immune dysregulation rather than virus replication is responsible for the increasing changes in vascular permeability associated with HCPS [23,24]. Upon hantavirus infection, the pathogens-associated molecular patterns (PAMPs) are recognized by the extracellular or intracellular receptors of endothelial host cells, leading to the local production of pro-inflammatory cytokines and chemokines, such as IL-1β, TNF-α, and IL-6, by activated macrophages [10,25,26]. Additionally, Th1 cells produce IFN-γ and TGF-β, cytokines that are responsible for cell-mediated immunity, regulated by IL-12 [10,27]. The regulatory T cells that produce the immunosuppressive cytokines IL-10 and TGF-β play an important role in the regulation of the immune response and limit the immunopathology induced by hantavirus infection [10]. Thus, we were interested in determining whether the levels of serum cytokines in patients at the time of their admission to hospital were associated with the final clinical outcome of the ANDV-induced disease. To do so, we compared the levels of serum cytokines in three distinct groups of individuals: non-infected individuals (healthy controls), ANDV-infected patients with a mild disease progression and ANDV-infected patients with a severe disease progression.

When studying IL-1β we found that the serum levels of this cytokine remain unaltered in ANDV-infected patients when compared to that of the non-infected controls (Fig 1A). This finding confirms previous reports showing no increase in the levels of IL-1β in patients infected with PUUV or in patients with HCPS [5]. Nonetheless, other studies show that the cells producing IL-1β and other pro-inflammatory cytokines are concentrated in the lung tissue of individuals with HCPS [26,28]. Therefore, these observations suggest that production of local cytokines in the lungs of ANDV-infected patients could differ from circulating levels of cytokines.

Results regarding the levels of IL-21 in hantavirus-infected individuals are dissimilar between previous reports [5,29]. One study shows a high overexpression of IL-21 in patients with hemorrhagic fever with renal syndrome (HFRS) caused by hantavirus [29]. In that study, the authors establish an association between the levels of IL-21 and the severity of HFRS. However, another study reports that there is no change in the levels of IL-21 in the serum of HCPS patients [5]. In contrast to both previous studies [5,29], our results show that in the group of ANDV-infected patients, IL-21 levels are significantly lower when compared to non-infected controls (p = <0.0001) (Fig 1C). It is worth noting that the pathology induced by New-World hantaviruses, HCPS, shares many, although not all of the clinical features of the disease caused by Old-World hantaviruses, HFRS. Thus, results obtained in the context of ANDV infection are not expected to fully match those that have been described for Old-World hantaviruses.

An interesting, yet expected finding, is that in ANDV-infected patients the serum levels of IL-12p70 are significantly lower (p = <0.0001) when compared to the non-infected control group. This observation is in full agreement with a study showing a similar trend in Dobrava virus (DOBV) infected patients [15]. A plausible explanation is to consider that the overexpression of a repressor cytokine, such as IL-10, would down regulate the levels of IL-12p70 [30]. This possibility is strongly supported by our results, in which the level of IL-10 in serum of ANDV-infected patients is significantly higher (p = <0.0001) than in the healthy controls (Fig 2C; Table 3). It is well known that the overexpression of IL-10 is strongly favored by high levels of IL-6 and TNF-α [15]. Additionally, overexpression of TNF-α and IL-6 are associated with inflammatory systemic disease, an increase in vascular permeability and with the production of a negative inotropic effect [31,32]. In agreement with this positive loop of activation, our results show that the levels of IL-6 and TNF-α are elevated in ANDV-infected patients (Fig 2A and 2D). In fact, when compared to non-infected controls, significant increases in the levels of TNF-α, IFN-γ, IL-10 and IL-6 in ANDV-infected patient are observed (p = <0.0001; 0.0036; <0.0001; <0.0001, respectively) (Fig 2A–2D). These findings are in full agreement with most previous studies [5,10,14,15,33], although TNF-α expression is not always raised in patients with severe HCPS [10]. This discrepancy cannot be readily explained; however, one possibility is to consider the genetic differences between the subjects included in these two studies, one conducted in Brazil [10] and the other in Chile. Differences between both populations have been previously reported in other studies [18,34].

Additionally, we compared TNF-α, IL-10, and IL-6 levels between mild and severe cases of ANDV-infection. Even though no significant differences in TNF-α or IL-10 levels exist between these two groups (Fig 2A and 2C, and Table 3), the IL-6 levels in the serum of ANDV-infected patients with a mild disease progression increased by 2.49 fold (p = 0.0021) compared to the non-infected controls (Fig 2D), whilst those with severe symptoms displayed a 100.7 fold (p = <0.0001) increase in the levels of IL-6 compared to the control group (Table 3 and Fig 2D). Moreover, when categorizing patients into survivors and fatalities the IL-6 levels in fatal cases of ANDV infection were significantly higher compared to survivor or control patients (Fig 3 and Table 4). Finally, a multivariate stepwise forward and reverse logistic regression model was constructed, confirming the relevance of the IL-6 levels at the time of patient hospitalization in the prediction of the outcome of HCPS (Table 5). It is well documented that IL-6 levels are significantly elevated in the serum of patients with HCPS, epidemic nephropathy (EP), and HFRS [5,12,35]. Consistent with our findings, other reports show that IL-6 levels are even higher in severe-disease patients compared with mild-disease patients [13,35]. Thus, our findings establish a clear association between the Th1-type cytokine IL-6 and the severity of ANDV-induced HCPS, suggesting that the serum concentration of IL-6 at the time of hospitalization can potentially be used as a molecular marker to predict the clinical outcome in ANDV-infected patients, offering the promise of personalized intervention, right from the moment of hospital admission.

## References

[1] Martinez VP, Bellomo C, San Juan J, Pinna D, Forlenza R, et al (2005) Person-to-person transmission of Andes virus. Emerg Infect Dis 11: 1848–1853. doi: 10.3201/eid1112.050501 16485469 10.3201/eid1112.050501 PMC3367635

[2] Padula PJ, Edelstein A, Miguel SD, Lopez NM, Rossi CM, et al (1998) Hantavirus pulmonary syndrome outbreak in Argentina: molecular evidence for person-to-person transmission of Andes virus. Virology 241: 323–330. doi: 10.1006/viro.1997.8976 9499807 10.1006/viro.1997.8976

[3] Enria DA, Briggiler AM, Pini N, Levis S (2001) Clinical manifestations of New World hantaviruses. Current topics in microbiology and immunology 256: 117–134. 11217400 10.1007/978-3-642-56753-7_7

[4] Figueiredo LT, Souza WM, Ferres M, Enria DA (2014) Hantaviruses and cardiopulmonary syndrome in South America. Virus Res 187: 43–54. doi: 10.1016/j.virusres.2014.01.015 24508343 10.1016/j.virusres.2014.01.015

[5] Morzunov SP, Khaiboullina SF, St Jeor S, Rizvanov AA, Lombardi VC (2015) Multiplex Analysis of Serum Cytokines in Humans with Hantavirus Pulmonary Syndrome. Frontiers in immunology 6: 432 doi: 10.3389/fimmu.2015.00432 26379668 10.3389/fimmu.2015.00432 PMC4553709

[6] Dolgin E (2012) Hantavirus treatments advance amidst outbreak in US park. Nat Med 18: 1448 doi: 10.1038/nm1012-1448a 23042337 10.1038/nm1012-1448a

[7] Jonsson CB, Hooper J, Mertz G (2008) Treatment of hantavirus pulmonary syndrome. Antiviral Res 78: 162–169. doi: 10.1016/j.antiviral.2007.10.012 18093668 10.1016/j.antiviral.2007.10.012 PMC2810485

[8] Macneil A, Nichol ST, Spiropoulou CF (2011) Hantavirus pulmonary syndrome. Virus Res 162: 138–147. doi: 10.1016/j.virusres.2011.09.017 21945215 10.1016/j.virusres.2011.09.017

[9] Bondu V, Schrader R, Gawinowicz MA, McGuire P, Lawrence DA, et al (2015) Elevated cytokines, thrombin and PAI-1 in severe HCPS patients due to Sin Nombre virus. Viruses 7: 559–589. doi: 10.3390/v7020559 25674766 10.3390/v7020559 PMC4353904

[10] Borges AA, Campos GM, Moreli ML, Moro Souza RL, Saggioro FP, et al (2008) Role of mixed Th1 and Th2 serum cytokines on pathogenesis and prognosis of hantavirus pulmonary syndrome. Microbes and infection / Institut Pasteur 10: 1150–1157. 10.1016/j.micinf.2008.06.006 18606242

[11] Kyriakidis I, Papa A (2013) Serum TNF-alpha, sTNFR1, IL-6, IL-8 and IL-10 levels in hemorrhagic fever with renal syndrome. Virus research 175: 91–94. doi: 10.1016/j.virusres.2013.03.020 23603136 10.1016/j.virusres.2013.03.020

[12] Linderholm M, Ahlm C, Settergren B, Waage A, Tarnvik A (1996) Elevated plasma levels of tumor necrosis factor (TNF)-alpha, soluble TNF receptors, interleukin (IL)-6, and IL-10 in patients with hemorrhagic fever with renal syndrome. The Journal of infectious diseases 173: 38–43. 8537680 10.1093/infdis/173.1.38

[13] Makela S, Mustonen J, Ala-Houhala I, Hurme M, Koivisto AM, et al (2004) Urinary excretion of interleukin-6 correlates with proteinuria in acute Puumala hantavirus-induced nephritis. American journal of kidney diseases: the official journal of the National Kidney Foundation 43: 809–816. 15112171 10.1053/j.ajkd.2003.12.044

[14] Sadeghi M, Eckerle I, Daniel V, Burkhardt U, Opelz G, et al (2011) Cytokine expression during early and late phase of acute Puumala hantavirus infection. BMC immunology 12: 65 doi: 10.1186/1471-2172-12-65 22085404 10.1186/1471-2172-12-65 PMC3259039

[15] Saksida A, Wraber B, Avsic-Zupanc T (2011) Serum levels of inflammatory and regulatory cytokines in patients with hemorrhagic fever with renal syndrome. BMC infectious diseases 11: 142. 21605369 10.1186/1471-2334-11-142 PMC3114737

[16] Outinen TK, Makela SM, Ala-Houhala IO, Huhtala HS, Hurme M, et al (2010) The severity of Puumala hantavirus induced nephropathia epidemica can be better evaluated using plasma interleukin-6 than C-reactive protein determinations. BMC infectious diseases 10: 132 doi: 10.1186/1471-2334-10-132 20500875 10.1186/1471-2334-10-132 PMC2885391

[17] Sundstrom KB, Nguyen Hoang AT, Gupta S, Ahlm C, Svensson M, et al (2016) Andes Hantavirus-Infection of a 3D Human Lung Tissue Model Reveals a Late Peak in Progeny Virus Production Followed by Increased Levels of Proinflammatory Cytokines and VEGF-A. PloS one 11: e0149354 doi: 10.1371/journal.pone.0149354 26907493 10.1371/journal.pone.0149354 PMC4764364

[18] Angulo J, Pino K, Echeverria-Chagas N, Marco C, Martinez-Valdebenito C, et al (2015) Association of Single-Nucleotide Polymorphisms in IL28B, but Not TNF-alpha, With Severity of Disease Caused by Andes Virus. Clinical infectious diseases: an official publication of the Infectious Diseases Society of America 61: e62–69. 26394672 10.1093/cid/civ830 PMC4657541

[19] Lee HW, Calisher C, Schmaljohn C, editors (1999) Manual of hemorrhagic fever with renal syndrome and hantavirus pulmonary syndrome.

[20] Padula PJ, Rossi CM, Della Valle MO, Martinez PV, Colavecchia SB, et al (2000) Development and evaluation of a solid-phase enzyme immunoassay based on Andes hantavirus recombinant nucleoprotein. J Med Microbiol 49: 149–155. doi: 10.1099/0022-1317-49-2-149 10670565 10.1099/0022-1317-49-2-149

[21] Medina RA, Torres-Perez F, Galeno H, Navarrete M, Vial PA, et al (2009) Ecology, genetic diversity, and phylogeographic structure of andes virus in humans and rodents in Chile. J Virol 83: 2446–2459. doi: 10.1128/JVI.01057-08 19116256 10.1128/JVI.01057-08 PMC2648280

[22] Marsac D, Garcia S, Fournet A, Aguirre A, Pino K, et al (2011) Infection of human monocyte-derived dendritic cells by ANDES Hantavirus enhances pro-inflammatory state, the secretion of active MMP-9 and indirectly enhances endothelial permeability. Virology journal 8: 223 doi: 10.1186/1743-422X-8-223 21569520 10.1186/1743-422X-8-223 PMC3104372

[23] Khaiboullina SF, Morzunov SP, St Jeor SC (2005) Hantaviruses: molecular biology, evolution and pathogenesis. Current molecular medicine 5: 773–790. 16375712 10.2174/156652405774962317

[24] Maes P, Clement J, Gavrilovskaya I, Van Ranst M (2004) Hantaviruses: immunology, treatment, and prevention. Viral Immunol 17: 481–497. doi: 10.1089/vim.2004.17.481 15671746 10.1089/vim.2004.17.481

[25] Markotic A, Hensley L, Daddario K, Spik K, Anderson K, et al (2007) Pathogenic hantaviruses elicit different immunoreactions in THP-1 cells and primary monocytes and induce differentiation of human monocytes to dendritic-like cells. Collegium antropologicum 31: 1159–1167. 18217475

[26] Mori M, Rothman AL, Kurane I, Montoya JM, Nolte KB, et al (1999) High levels of cytokine-producing cells in the lung tissues of patients with fatal hantavirus pulmonary syndrome. The Journal of infectious diseases 179: 295–302. doi: 10.1086/314597 9878011 10.1086/314597

[27] O'Shea JJ, Ma A, Lipsky P (2002) Cytokines and autoimmunity. Nature reviews Immunology 2: 37–45. doi: 10.1038/nri702 11905836 10.1038/nri702

[28] Kilpatrick ED, Terajima M, Koster FT, Catalina MD, Cruz J, et al (2004) Role of specific CD8+ T cells in the severity of a fulminant zoonotic viral hemorrhagic fever, hantavirus pulmonary syndrome. Journal of immunology 172: 3297–3304. 10.4049/jimmunol.172.5.3297 14978138

[29] Chen H, Liu H, Wang Y, Yang Y, Zhao Y (2014) Elevated serum IL-21 levels in hantavirus-infected patients correlate with the severity of the disease. Inflammation 37: 1078–1083. doi: 10.1007/s10753-014-9831-3 24510007 10.1007/s10753-014-9831-3

[30] de Waal Malefyt R, Abrams J, Bennett B, Figdor CG, de Vries JE (1991) Interleukin 10(IL-10) inhibits cytokine synthesis by human monocytes: an autoregulatory role of IL-10 produced by monocytes. The Journal of experimental medicine 174: 1209–1220. 1940799 10.1084/jem.174.5.1209 PMC2119001

[31] Finkel MS, Oddis CV, Jacob TD, Watkins SC, Hattler BG, et al (1992) Negative inotropic effects of cytokines on the heart mediated by nitric oxide. Science 257: 387–389. 1631560 10.1126/science.1631560

[32] Meldrum DR (1998) Tumor necrosis factor in the heart. The American journal of physiology 274: R577–595. 9530222 10.1152/ajpregu.1998.274.3.R577

[33] Krakauer T, Leduc JW, Krakauer H (1995) Serum levels of tumor necrosis factor-alpha, interleukin-1, and interleukin-6 in hemorrhagic fever with renal syndrome. Viral immunology 8: 75–79. doi: 10.1089/vim.1995.8.75 8825292 10.1089/vim.1995.8.75

[34] Borges AA, Donadi EA, Campos GM, Moreli ML, de Sousa RL, et al (2010) Association of -308G/A polymorphism in the tumor necrosis factor-alpha gene promoter with susceptibility to development of hantavirus cardiopulmonary syndrome in the Ribeirao Preto region, Brazil. Arch Virol 155: 971–975. doi: 10.1007/s00705-010-0655-7 20372945 10.1007/s00705-010-0655-7

[35] Fan W, Liu X, Yue J (2012) Determination of urine tumor necrosis factor, IL-6, IL-8, and serum IL-6 in patients with hemorrhagic fever with renal syndrome. The Brazilian journal of infectious diseases: an official publication of the Brazilian Society of Infectious Diseases 16: 527–530. 10.1016/j.bjid.2012.10.002 23141988

